# Supplementary material for: ‘The illness isn’t the end of the road’—Patient perspectives on the initiation of and early participation in a multi-disease, community-based exercise programme
Source: PLoS One. 2024 Mar 29;19(3):e0291700. doi: 10.1371/journal.pone.0291700 (PMC10980187; doi:10.1371/journal.pone.0291700)
Supplement: S2 Appendix — (DOCX) [file pone.0291700.s002.docx]

## S2 Appendix. Topic guide followed by moderator in each focus group

| Topic | Typical Questions • Probes |
| --- | --- |
| Journey to MCEP | What was your initial perception of coming down to MedEx?  When the programme was suggested to you, how did the idea strike you?   - What influenced you to join the programme? - Did you have a clear understanding what you were signing up for? - What aspects might have worried or concerned you? |
| Experience of the programme | Now that you are here what has been your experience of the programme?  Looking at all aspects, the programme design, the referral from the hospital, the social side, the education talks |
| The exercise class | What aspects did you like/dislike?  Suitability of classes  Looking at the running, structure, content of the exercise class |
| Factors that facilitated participation in the programme | What has helped your participation in the programme?  Who has helped?   - What did the person/people do to support you that made it effective?   Were there any supports that were good/you think are needed?   - At what stage do you feel support is needed most and why? - Are there more ways that people referred to the programme could be supported?   Any barriers you felt you had to overcome? |
| Perceived benefits of the MCEP setting | What effect do you think the programme has had on you?   - Social/Mental/Physical - Any changes in amount of PA you partake in weekly? |
| Recommended improvements | What improvements would you suggest to this programme?   - Consider referral process/ the classes/ the talks/ the gatherings/ the initial assessments/ any other factors. |
