# Supplementary material for: ‘The illness isn’t the end of the road’—Patient perspectives on the initiation of and early participation in a multi-disease, community-based exercise programme
Source: PLoS One. 2024 Mar 29;19(3):e0291700. doi: 10.1371/journal.pone.0291700 (PMC10980187; doi:10.1371/journal.pone.0291700)
Supplement: S3 Appendix — (DOCX) [file pone.0291700.s003.docx]

## S3 Appendix. Themes, subthemes and supporting quotes

| **Theme** | **Subtheme** | **Supporting Quotes** |
| --- | --- | --- |
| Moving from fear to confidence | Fear and uncertainty | ‘*I’ve been lacking confidence in doing exercises …. I was wondering would it bring on the pain, the angina I had… I didn’t know where I was going’* (P6 FG1)  ‘*Fear is the worst thing because you are on your own and you are just wondering am I able to do it or am I not able to do it* [exercise]*’* (P7, FG1)  *‘Yes, fear is the thing, in case we over do it.*’ (P3 FG1)  ‘*we were afraid to do anything’* (F1 FG3) (a number more agreeing in the background)  *‘I think we all had that feeling that we were afraid to do anything’* (F3 FG3)  *‘once this* [the cardiac event] *happened, I was afraid to walk – there was weeks I didn’t go out because I thought oh no….’* (F1 FG3)  *‘when you have a major episode in life, quite a lot gets stolen away from you. You live with the trauma of what you have gone through …. We were all nervous about what you go through.’* (M3 FG4)  *‘thing for me is.... you have these operations in hospital, and I’m just talking about myself, but you have this pity for yourself.’ (*P6 FG1)  *‘You’re coming out of hospital; you’re painted as dead’* (M2 FG4)  *‘Before I wouldn’t know what exercises to do or anything like that.’* (F1 FG4)  *‘Well, I thought I mightn’t be able to do much, or I wouldn’t be able to manage it* [exercise]*’* (M1 FG4)  *‘I want to be able to use a chain saw again and to chop wood again, I really wasn’t fit for it.’ (*M3 FG4)  *‘sure, I wouldn’t know what I needed to do’* (M3 FG4)  ‘*I felt after it* [hospital-based CR] *finished I didn’t know where to go’* (M1 FG3)  ‘*you’re down doing the exercise in the rehab* [hospital] *and when that was over that was it, you were gone’* (M4 FG3).  *‘I would never have warmed up before. I would have just started walking with a lot of pain in my shins. So, I thought that was great.’* (P10, FG2)  *‘I wouldn’t have known about the warmup or the cool down.’* (P9, FG2) |
|  | Need for continuity | *‘...it was something we were waiting for, all of us’* (P1 FG1)  *‘they (hospital) rang me up and sent me out a letter and so I’m doing it now.*’ (P12 FG2)  *‘I did the CR programme in the hospital, and they had mentioned it up there.’ (P8 INT 1)*  *‘They (the hospital) told me about it’ (F1 FG4)*  *‘Audrey (senior physio) would have told us about it now’ (F4 FG3)*  *‘It was pretty much straight forward, she (senior physio) just told us in the rehab class, and we came down here’ (M1 FG3)*  *‘If you were worried [medical person] was there to reassure us.’* (P3, FG1)  *‘It’s having someone you know and who knows you’* (M3, FG2).  *‘The continuity and the link between the hospital and yourselves* [the programme] *is critical, it’s really important.’* (M3, FG4)  *‘You’ve a back-up really, any problem you just talk to …. and they refer you back. It’s knowing you can get back to them’ (F2, FG3)*  *‘We’ve got that link. We don’t want to lose that support now that we have it.’ (M1, FG3)*  *‘You’d walk all right but you wouldn’t do the exercises* [circuit]’ (F2 FG 3)  *‘I was told to get out walking and that didn’t really suit me as I was a bit of a crock so I got on a bicycle and I found that a great alternative, I can do that 3 -4 days a week and then 2 days here so as you said the discipline of getting up and getting out’ (M3 FG3)*  *‘otherwise I wouldn’t go to the gym myself… I was delighted to get the chance’* (F2, FG4)  *‘It was the first time I have been to the gym and had to use the machines and not be afraid.’* (F2 FG4)  ‘*Also, you learn how to do exercises. Before I wouldn’t know what exercises to do or anything like that and it’s great to be able to do them at home now that I understand what I should and shouldn’t do.*’ (F1 FG4)  *‘You see those gym things, I would have never walked into a gym, I had never been in a gym…… Everyone seemed to like the gym, you would feel really awkward going in, you wouldn’t know what you were doing before that. At least now when I sit down, I know what I’m supposed to be doing.’* (P10, FG2)  *‘You certainly wouldn’t have had the confidence to go in and use those machines* [before the programme]’ (P7, FG1) |
|  | Increase in confidence | *‘you actually feel safe in the environment which is supervised, they are professionals, and nothing is going to happen. But if something did happen, they are the right people to deal with it’ (P2, FG1)*  *‘The combination of all of them was very beneficial.’ (P3, FG1)*  *‘It was a team effort’ (P7, FG1)*  *‘…you trusted the staff that were looking after us, you knew that they were not going to put you in harm’s way, they are there to take care of you.’ (P7 FG1)*  *‘They do a 3 stage of what you can do yourself, you’re not being pushed into working flat out, you do what you can’ (M2 FG4)*  *‘they [the instructors] are very good at that and they also tell us not to overdo it – gives you direction.’ (M1 FG3)*  *‘they were always reassuring us that if we had any problems, to stop. Not to overdo it.’ (P1 FG1)*  *‘The instructors encouraged you, but you weren’t pushed. If you weren’t able to do something, that was ok.’ (P9, FG2)*  *‘They step out in front of us, and we do it, then they walk around and look back to see what we are doing and see are you doing the right thing.’ (F2 FG4)*  *‘We thought we couldn’t do* [the exercises] *and now we can do everything….’* (F2 FG3)  *‘More confidence anyway in yourself you know – you were afraid to do anything in case you were doing too much or too little you know so you have that confidence that you know you able to do a lot more.’* (F2 FG3)  *‘I have to say I feel better …. because I'm more confident in myself’* (P6 FG1)  ‘*my daughter has a treadmill at home, and I was afraid to go on it, but I go on it now you know’* (F2 FG4)  *‘Before I wouldn’t know what exercises to do or anything like that and it’s great to be able to do them at home, now that I understand what I should and shouldn’t do’* (F1 FG4)  *‘Some of us are coming back here next week* [outside of the programme] *and we are just going to go the gym.*’ (P1 FG1) |
|  | Life beyond illness | *‘Absolutely beneficial, in every way. It was something we looked forward to and was excited to see who was going to take part after all, we are delighted…. We have really enjoyed it; we have got the bug…wonderful experience.’* P3 FG1  *‘.. they don’t treat us as recovering patients, and you’re no longer a patient. That’s a huge thing …. All from different backgrounds …. and it’s by being targeted normally that you’re well able to do this….* (M3 FG 4)  *‘That* [their condition] *would rarely come up. Very, very rarely.’* (F1 FG4)  ‘*Well, that’s in the background* [being a patient], *it’s gone*.’ (F2 FG4)  *‘…the illness isn’t the end of the road…’* (M2 FG3) |
| Drivers of engagement | Scheduled exercise | *‘I would have done none [exercise], literally none.’ (P9, FG2).*  *‘It's fantastic, just fantastic. Because you will not do it at home, you will not do it by yourself.’ (P1 FG1)*  *‘Well, I have an exercise bike, I used to try but not much……. And the longer you leave it, the less you are inclined to do something. It [the programme] was great……..you come in and do a class and you walk out the door and feel completely different, instead of sat at home giving out to yourself for not doing it.’ (P10, FG2)*  *‘You wouldn’t do it otherwise; you wouldn’t do it at home’ (F1 FG3)*  *‘If we hadn’t this [the programme] we’d be home sitting on the couch.’ (F1 FG4)*  *‘You see what happens is you go and have heart problems and you get it sorted out and you go to the cardiac rehab up in Sligo hospital, which is very good. You get hooked up to machines and everything but then you get sent home and you're told carry on walking with this and within a month you go back to your normal self and you're not doing anything.’ (P1 FG1)*  *‘You know you had two dates in the week you had to meet and otherwise you might have done nothing.’ (P8, Int. 1)*  *‘The discipline part of it is fantastic that you make yourself come twice a week’ (F2, FG3)*  *‘at least them 2 days, that you are sure of doing your exercise …. those 2 days you’re committed, you know what you’re doing and you’re going to go…… some of us but not all mightn’t be as good at doing it at home.’ (F2, FG4)* |
|  | Social connections | *‘I heard about this from my sister-in-law so I rang up to see could I join up’ (M3 FG3)*  *‘Anyone I talked to recommended it highly.’ (F1, FG4)*  *‘I know of a girl who already was coming to this session …. That’s where I heard about it first …. Then I asked … about it’* (F2 FG4)  ‘*Participant X was present. He went through it, and he advised me to do it.’* (M3 FG4)  *‘everyone’s in the same boat’ (M2C FG4)*  *‘all on the same wavelength’ (F1C FG4)*  *‘We are more comfortable talking with each other.’ (P4 FG1)*  *‘And then the social aspect of it too. In the rehab programme in the hospital, once you are finished with it, you are gone out the door straight away. This is nicer where you get to meet a few people, and I can talk to people who have similar issues and on similar medications to myself.’ (P8, INT 1)*  *‘I felt this was great because we bounce off each other, meet people with the same situations that we have all been through and psychologically it was a chance to meet other people, talk…’ (M1B FG3)*  *‘…. fantastic therapy …… you come down here and you think everyone has had stents put in but it’s not, people have had different problems and you just start talking to people and it makes people at ease more…’ (M4 FG3)*  *‘I’m not as bad as I thought I was [laughing] I’ve only 2 stents...’ (M3 FG3)*  *‘...we were counting who had the more stents at this stage you know we found out there’s always someone better or worse than you’ (M1, FG3)*  *‘...well, if they can do it so can I.’ (P2 FG1)*  *‘You see new people coming in now and they aren’t able to do what we can do, and we were once them…. and we see them kind of struggling and we say don’t try to do what we are doing because you won’t be able for it …’ (M1 FG3)*  *‘…the chat can be about anything.’ (M2 FG4).*  *‘After my open-heart surgery, I had a problem with the stitches down where the wound was and a lot of the vest that you buy have a hem which was rubbing against the wound irritating it. And I discovered [through talking to someone during the tea and chat] if I turned it inside out it made a big difference, and it was ages, absolute torture trying to figure it out.’ (FG1 P7)*  *‘And we talk among ourselves about what we’re eating and what we’re not eating and what suits us and what doesn’t and it’s the social gathering as well.’ (M1 FG3)*  *‘centre piece of this whole thing.’ (M3 FG4)*  *‘It was the motivational encouragement you got from others [in the group]’ (FG1 P3)* |
|  | Enjoyment | *‘We had fun too, a lot of laughter and that’s very important too’ (P3 FG1)*  *‘…we actually have fun…. who would associate P.E. and fun!’ (M3 FG4)*  *‘there’s a great fun aspect to it also – we have great fun down there [circuit class]’ (F1 FG3)*  *‘The wit and the banter that goes on – makes it for everybody’ (M2 FG3)*  *‘… try to do an exercise and no music, it’s totally different, meaningless.’ (F2 FG3)*  *‘Yes, the music and the exercise yes and it makes it more interesting.’ (M1 FG4)*  *‘If the music is off there’s something wrong.’ (M2 FG4)*  *‘I thought the gym was the best’ (P12 FG2)*  *‘I liked getting back into the gym. Before that it was circuit training in here [the studio], which was grand but after a few times doing it.... maybe if there was a wee bit more of the gym and wee bit less of the circuit training …. vary it and give you a bit more interest, you know…. It’s not like we didn’t like the circuit training classes it’s just if they were a bit more mixed.’ (P2 FG1)*  *‘I liked the combination of the circuit training and the gym.’ (P10 FG2)*  *‘I liked all the different exercises and we were not long doing them….The time went around quicker.’ (P6 FG1)*  *‘I’d like a little bit more variety into it’ (F4 FG3)*  *‘vary it and give you a bit more interest, you know….’ (P2 FG1)* |
| Challenges to keeping it (exercise) up | Barriers | *‘Ideally, I would, but once I go back in September, I can’t see it happening. Can’t see myself having the time to do it.’* (P8, Int. 1)  *‘The only negative thing is that* *I hadn’t the time to do it all the time because I was working.’* (P10, FG2)  ‘*I couldn’t do it all the time because I was babysitting’* (P12, FG2)  ‘*Yeah, the group* [hospital CR] *that I was in were interested but it just didn’t suit their work.’* (P9, FG2)  *‘We lost a lot of people because it clashed with dropping off kids and grandkids’* (F1, FG4)  *‘As it’s ongoing, as the year gets darker it’s very early in the morning’* (F2, FG4)  *‘there’s one man that comes from Enniscrone and he’d have to leave at 7 o’clock to be here. Wouldn’t it be terrible if he’d to drop out because of this, I’d imagine other people dropping out too*’ M3 FG4 |
|  | Dependency | *It* [medical support] *was automatically there, we had the support, and it was always at the back of your mind, they are there and that is great.* (P2, FG1)  *‘she (senior physio) was there, you know on hand, to say I got a wee pain here what’s this all about, am I doing too much, am I going too quick, too slow, can I do more, and I thought that was very good to have somebody professional like herself here all the time, I know with staff shortages but if for future references that we could have someone here professional all the time to talk to that if we weren’t feeling 100% that she could give us directions’* (M1 FG3)  ‘*Yes regularly to be here’* (Requesting medical presence) (M2 FG3)  ‘*You’ve a back-up really, any problem you just talk to …. and they refer you back. It’s knowing you can get back to them’* (F2, FG3)  ‘*We’ve got that link. We don’t want to lose that support now that we have it.*’ (M1, FG3)  *‘If there was subliminal support every week, I’d be happy enough. Just to know that it’s there.’* (P8, Int. 1)  *‘you can actually see them going around to each individual and they pick out somebody who’s under stress and bring them out and measure their heart rate.*’ (M3 FG4)  *‘It* [blood pressure monitoring*] reassures you that you’re ok for it.*’ (F1, FG4)  *‘Getting your blood pressure taken … it keeps you focused on it, otherwise when would you have it taken…. it’s nice to know you’re plodding along nicely.’* (F4, FG3)  *‘they monitor you, they come around and take a heart rate.’* (F1, FG4) |
