# Supplementary material for: ‘The illness isn’t the end of the road’—Patient perspectives on the initiation of and early participation in a multi-disease, community-based exercise programme
Source: PLoS One. 2024 Mar 29;19(3):e0291700. doi: 10.1371/journal.pone.0291700 (PMC10980187; doi:10.1371/journal.pone.0291700)
Supplement: Attachment — Submitted filename: Response to Reviewers.docx [file pone.0291700.s004.docx]

# Rebuttal Letter

Michael Thomas Lawless, Ph.D.
Academic Editor
PLOS ONE

Cambridge, United Kingdom

August 3^rd^, 2023

Dear Dr. Lawless,

**RE: Rebuttal Letter for PONE-D-23-24657**

‘The illness isn’t the end of the road’ - Patient perspectives on the initiation of and early participation in a multi-disease, community-based exercise programme.

Thank you for reviewing and forwarding feedback on the manuscript. We appreciate the opportunity to submit a revised draft of the manuscript for consideration. Please find included below responses to each point raised by the academic editors and reviewer(s). The following files have been uploaded.

1. ‘Revised Manuscript with Track Changes’

2. ‘Manuscript’ - an unmarked version

3. ‘Fig1.tif’ - image of flowchart requested

4. ‘S1_Appendix’ – file renamed

5. ‘S2_Appendix’ – file renamed

6. S3_Appendix – renamed and updated

**Journal Requirements:**

|  | **Academic editors’ comments** | **Response** |
| --- | --- | --- |
| 1. | Please ensure that your manuscript meets PLOS ONE's style requirements, including those for file naming. | We have reviewed and amended the manuscript in line with PLOS ONE’s style requirements as outlined in links attached |
| 2. | Consider depositing your raw data in a repository | Authors agree that this data is collected from a small group of participants and full transcripts should not be shared as they may involve indirect identifiers (such as sex, ethnicity, location, etc.) that may risk the anonymity of study participants.  However, all quotes extracted for data analysis are available in S3 Appendix. Themes, subthemes and supporting quotes. |
| 3. | Ethics statement should only appear in the Methods section of your manuscript | This has been checked and ethics statement appears in methodology only. It is now deleted from statements and declarations. |
| 4. | Update your Data Availability Statement | Data in Supporting Information Files.  The authors confirm that the data supporting the findings of this study are available within the article [and/or] its supplementary materials. |
| 5. | Review reference list to ensure that it is not retracted, complete and correct | All references have been checked as requested. Retraction was completed based on the following article Thielen J. When scholarly publishing goes awry: Educating ourselves and our patrons about retracted articles. portal: Libraries and the Academy. 2018;18(1):183-98. |

**Reviewers Comments:**

|  | **Reviewer #1 Specific Comments** | **Response** |
| --- | --- | --- |
| 1. | If data saturation was considered when determining the number of study participants? | Line inserted to support data saturation leading to sample size determination Line: 141-142 |
| 2. | If transcripts were returned to participants and/or if participants checked or provided comment on the themes? | The opportunity was not available to return transcripts to participants. |
| 3. | If software was used to analyse the data (such as Nvivo)? | Analysis was completed in Microsoft Word following the Braun and Clarke approach |
| 4. | The results section is very clear although I did wonder why some quotes were part of a sentence and others were indented and included on the next available line? | Amended the quotes according to the APA guidelines where quotations of fewer than 40 words are now in quotation marks within the text and quotation of 40 words or more are in block quotation indented below the text. This applies to the whole results section. Line 189-354 |
| 5. | Line 416 wasn't should be was not. | Amended, Now Line: 432 |
| 6. | Wondered if the exercise classes were going to be continued or not? and if this could be reported. | Yes, the MCEP was an ongoing programme with attendance encouraged beyond the 10 weeks – reported now in Line 112-113 |
|  | **Reviewer #2 Specific Comments** | **Response** |
|  | **Introduction** |  |
| 1. | Line 72-75: Please say what is the main barriers that only less than half receive CR? Specifically for women? (ie. REFS: https://doi.org/10.1016/j.cjca.2023.07.016 ; (Ghisi et al 2023) https://doi.org/10.3390/ijerph182413113 ; (Winnige et al 2021) https://doi.org/10.3390/ijerph20054064 ; (Antoniou et al 2023) | The literature referenced in the manuscript focuses on barriers and facilitators to adherence to maintenance CBCR programmes – follow on from phase II CR. Although, there are similarities with the barriers to Phase III CR reported in the references kindly provided by the reviewer, it was decided that the manuscript should retain its focus on the unique barriers to follow-on maintenance CBCR. |
|  | **Methodology** |  |
| 2. | Please define the way of samples’ selection. Was it random? Please describe the way of choosing them.  How was the sample size defined? | Purposive sampling was used based on those referred to the programme by SUH and still attending at 10 weeks – Clarified in Line 96-99  Sample size was determined by data saturation, the point when no new information was gained. Clarified in Line: 141-142 |
| 3. | Please say more about CR programme. Describe FITT parameters. What about those who was taking b-blockers? | Table 1 added in Line 128 and referred to in text in Line 117-118 describes the FITT prescription for the programme, including the approach taken for patients prescribed β-blockers. Rating of perceived exertion (RPE) was mainly used to measure intensity to mirror the protocol in the hospital-based CR at SUH. When HR was randomly checked, the target HR (THR) for those on beta blockers was reduced by 30bpm, with THR calculated to 40-70% of HR reserve (HRR)/60-80%HRmax. |
| 4. | Add a table with CR components. | Added table 1 – Line 128 |
| 5. | What are the primary and secondary outcomes? | In this qualitative research, the aim was to explore patients experiences of the initiation and early participation in a MCEP programme and the dimensions that facilitate and hinder physical activity engagement in individuals with CVD. Other research, to be published separately, evaluated the effect of the MCEP on cardiorespiratory fitness (primary outcome) and resting blood pressure and HR, body composition, upper and lower muscular strength, flexibility, and health related quality of life measurements (secondary outcome(s)). |
| 6. | Please add a flowchart. | We have added in Fig 1 Flowchart of eligible participants and recruitment see Line 173/ 175 and saved as a .tif file and uploaded to PACE |
|  | **Discussion** |  |
| 7. | What about safety? Does it affect participation? | Safety and its effect on participation is now addressed in results and discussion.  No serious adverse events reported and perceived safe environment referred to in Line 220-223 under results with new quote added in to support this. Also updated S3 Appendix. Themes, subthemes and supporting quotes.  Along with the inclusion that the participants viewed the MCEP as a safe environment to exercise under results Line 220-223, it is also part of discussion Line 369-370 |
| 8. | Please add a short discussion what would be the ideal dose of CR (how many weeks are enough?). There are several hundred references in the field of CR, in the last years. doi: 10.1136/heartjnl-2012-303055 (Sandercock et al 2013) | As this is a follow on to hospital-based CR the ideal dose is for the participants to reach their PA guidelines i.e. at least 150-300min of moderate intensity aerobic exercise per week and 2+ days of LME – this is discussed in Lines 377-381. This is now linked to the recommended reference (Line 381-382) highlighting that dose of exercise prescribed is important to meet PA guidelines and achieve meaningful benefits |
|  | **Future Research** |  |
| 9. | include in the limitations the strengths of this study, a justification that the search missed the field of telehealth interventions primarily based on a home-based approach and include keywords (telehealth, mhealth, telerehab). doi:10.7759/cureus.23485 (Nso et al 2022) <https://doi.org/10.1016/j.jbmt.2021.07.009> (Papera et al 2021) DOI:https://doi.org/10.1016/j.ijcard.2022.08.055 (Batalik et al 2022) | Included Nso et al 2022 in the discussion for the potential for telehealth to be incorporated to support participants in meeting their PA guidelines Line 384-386 linking it to potential future research. |

**Extra Comment at the end**

| 1. | While revising your submission, please upload your figure files to the Preflight Analysis and Conversion Engine (PACE) digital diagnostic tool, <https://pacev2.apexcovantage.com/>. PACE helps ensure that figures meet PLOS requirements. | Completed this for Fig 1. Flowchart of eligible participants and recruitment under [joanne.regan@atu.ie](mailto:joanne.regan@atu.ie) account on PACE |
| --- | --- | --- |

We hope you find the responses to all comments meet your satisfaction. Please contact us if you have any further recommendations or amendments you require.

Sincerely

Dr. Joanne Regan-Moriarty,

Department of Health and Nutritional Science, ATU Sligo, e-mail: joanne.regan@atu.ie

Corresponding Author

Prof Niall Moyna, DCU, Dept of Health and Human Performance, Dublin, e-mail : niall.moyna@dcu.ie
